# Versatility and Stereotypy of Free-Tailed Bat Songs

**DOI:** 10.1371/journal.pone.0006746

**Published:** 2009-08-25

**Authors:** Kirsten M. Bohn, Barbara Schmidt-French, Christine Schwartz, Michael Smotherman, George D. Pollak

**Affiliations:** 1 Department of Biology, Texas A&M University, College Station, Texas, United States of America; 2 Section of Neurobiology, University of Texas at Austin, Austin, Texas, United States of America; 3 Bat Conservation International, Austin, Texas, United States of America; University of Sussex, United Kingdom

## Abstract

In mammals, complex songs are uncommon and few studies have examined song composition or the order of elements in songs, particularly with respect to regional and individual variation. In this study we examine how syllables and phrases are ordered and combined, ie “syntax”, of the song of *Tadarida brasiliensis*, the Brazilian free-tailed bat. Specifically, we test whether phrase and song composition differ among individuals and between two regions, we determine variability across renditions within individuals, and test whether phrases are randomly ordered and combined. We report three major findings. First, song phrases were highly stereotyped across two regions, so much so that some songs from the two colonies were almost indistinguishable. All males produced songs with the same four types of syllables and the same three types of phrases. Second, we found that although song construction was similar across regions, the number of syllables within phrases, and the number and order of phrases in songs varied greatly within and among individuals. Last, we determined that phrase order, although diverse, deviated from random models. We found broad scale phrase-order rules and certain higher order combinations that were highly preferred. We conclude that free-tailed bat songs are composed of highly stereotyped phrases hierarchically organized by a common set of syntactical rules. However, within global species-specific patterns, songs male free-tailed bats dynamically vary syllable number, phrase order, and phrase repetitions across song renditions.

## Introduction

The use of vocal displays by animals to defend territories and attract females is widespread [1], [2]. Among vertebrates these vocalizations most often take the form of simple repetitions of one or a few syllables and are generally referred to as mating or advertisement “calls”. In a few exceptional cases, such as songbirds [3], bats [4], [5] and whales [6], these advertisement signals can be more complex vocalizations termed “songs”. The major difference between mating “calls” and “songs” is that songs are longer and contain multiple types of elements (e.g. syllables, notes and/or phrases) that are often combined in a stereotypical manner [7], [8]. In most songs, element orders are not random, but are instead highly structured, with individual, regional, and/or species-specific patterns [8]–[11]. Therefore, songs have an added structural dimension in the form of “syntax”- the patterns by which elements are ordered and combined. Songs are often hierarchically organized where notes are combined into syllables, syllables into motifs and motifs into phrases with multiple layers of repetition or periodicities [7], [8]. While these features are common in birds, evidence of hierarchical syntax in mammals is scarce.

For most birds the production of calls and simple songs is largely innate, but a subgroup of birds known as the vocal learners, which includes the parrots, hummingbirds and oscine songbirds, are endowed with a specialized network of brain nuclei that constitute a “song system” allowing them to produce broad repertoires of songs that are more complex and flexible than the songs of other birds [12]. Thus, among birds song complexity varies and the degree of complexity is correlated with the sophistication of the underlying vocal control circuitry of the brain [12].

Singing behavior and examples of vocal syntax are exceedingly rare in mammals. Simple songs resembling the innate songs of non-vocal learning birds have been observed in mice *Mus musculus*, [13] and more elaborate examples of singing behavior have been identified in primates [14], [15], cetaceans [16] and bats [4], [5], [17]. However, unlike the large body of literature on bird song syntax, mammalian research has rarely gone beyond determining that the order of song elements is non-random [13], [16], [18]. Few studies have identified species-specific rules for element orders or examined variation in song syntax with respect to individuals or regions. Although there have been reports on courtship songs in bats [17], the extent to which bat songs are syntactically organized is unknown and virtually nothing is known about how much control bats have over the hierarchical organization of their songs.

**Figure 1:**
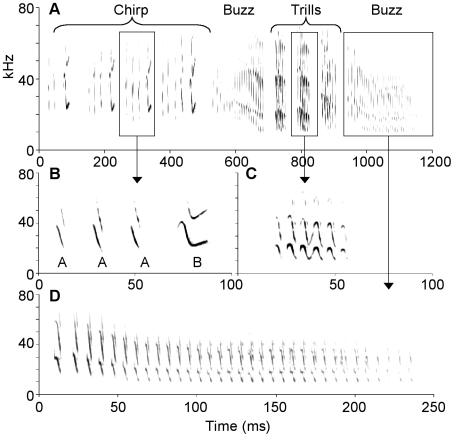
Structure of *T. brasiliensis* song. One complete song showing the three types of phrases: chirp, trill, and buzz (A). Expanded section of a chirp phrase showing one motif which is composed of two types of syllables: type A and type B (B). Expanded section of a trill (C). Expanded section of a buzz (D). This song is a chirp-buzz-trill-buzz song type.

In this paper we examine composition and phrase order, ie “syntax” of male Brazilian free-tailed bat song (*Tadarida brasiliensis*). The Brazilian free-tailed bat is an abundant and genetically contiguous subspecies occurring from California to Mexico [20]. During the mating season males establish territories, which they vigorously and aggressively defend against encroaching males, but they allow multiple females to enter and reside in their territories. During this period songs are easily evoked from territorial males when either males or females approach their territories [4], [21] (See Movie S1 and Movie S2). *T. brasiliensis* songs are complex vocal signals with multiple types of syllables and phrases (Fig. 1). Phrases are highly stereotyped, discrete, and distinct, and as such provide an excellent model for examining how elements are combined in a composite mammalian vocal signal. In this report, we examine regional and individual song variation and stereotypy by comparing vocalizations across individuals and between two different locations: a captive colony in Austin and a natural colony in College Station, Texas. In addition, we examine how phrases are ordered. We test whether phrase order deviates from random models and then use deviations from random predictions to identify specific syntactical rules for song construction.

## Results

### Song Characteristics

We examined 319 songs from 17 bats in Austin and 93 songs from 16 bats in College Station, for a total of 412 songs from 33 bats. The songs of *T. brasiliensis* are composed of up to three types of phrases that are easily recognized: chirps, trills and buzzes (Fig. 1). Chirps are complex phrases composed of two types of syllables: “A” and “B” syllables. “A” syllables are short (mean = 5 ms) downward frequency modulated (FM) sweep syllables (Fig. 1b). Previous research has shown that B syllables are longer (mean = 17ms) and more complex than A syllables [4]. B syllables often begin with an upward FM followed by a longer downward FM and some signals end with a second upward FM. Thus, their spectral contours often have multiple inflection points. Each B syllable is often preceded by A syllables (80% of 2,247 B syllables preceded by between 1 and 24 A syllables) and the sequence of A syllables followed by a B syllable is then repeated to form the chirp phrase (range 1–29 repeats).

The second type of phrase is the trill. Trills are composed of short (mean = 3.4 ms), downward FM syllables that can be connected, resulting in sinusoidal patterns (Fig. 1c). Trill syllables, whether discrete or connected, are produced as a distinct phrase or burst with durations of approximately 25 ms (mean: 24±0.9 ms *N* = 70, range 8–45 ms) and have average intervals between syllables of 3.7 ms [4]. Although approximately 37% of trills were followed by another trill (see *Phrase Order* below), each trill was highly distinctive since each phrase was separated from the next by a silent interval of on average 35.6±0.6, (*N* = 127, range 20–60 ms) that is much greater than, and did not overlap with the duration of intervals between syllables within each phrase (maximum interval within a phrase = 14 ms).

The third phrase in song is the buzz. Buzzes are also composed of short (mean = 3 ms) downward FM syllables (Fig. 1d), but the syllables are never connected. Instead they are always separated by a few ms, mean = 4.4 [4]. Although the acoustical structure of trill and buzz syllables are similar, phrases can be differentiated by two features. The first is the number of syllables in a phrase. Trills have on average 4.1±0.2 syllables whereas buzzes have on average 35.0±2.7 syllables. This difference in syllable count was highly significant (paired t-test: *t* = 11.6, *P*<0.0001, *df* = 22). Due to the greater number of syllables in buzzes compared to trills, buzzes have substantially longer durations (buzz duration 241.7±16.6, paired t-test *t* = 12.8, *P*<0.0001, *df* = 22). The second feature that distinguishes buzzes from trills is that the spectral structure of successive buzz syllables follows a pattern [22]. The initial FM syllables in each buzz have relatively high beginning and end frequencies and are followed by 5–10 syllables with progressively lower beginning and end frequencies. The progressive decrease in end frequencies stabilizes after the first few syllables while the beginning frequencies continue to decrease resulting in smaller bandwidths (Fig. 1d). Like trills, two or more buzzes were produced sequentially (40% of transitions, see *Phrase Order* below), but were highly distinctive; each buzz phrase was separated from the following buzz by a relatively long silent interval (mean: 40.8±0.81, *N* = 113, range 25–85 ms) that was much greater than, and does not overlap with the interval between syllables within phrases (maximum interval within a phrase = 16 ms). We point out that while some songs contained all three types of phrases, other songs had only one or two of the possible phrase types.

In the above section we described the various syllables and phrases of the songs of male *T. brasiliensis*. Below we evaluate the composition and temporal patterns of songs in greater depth. In the first section we describe songs recorded in Austin and College Station, identify syllables and phrases that are shared by both colonies and test whether song composition differs between the two locations. In the second section we examine variation of song features in individual bats to determine the degree to which songs vary from one rendition to the next. Finally, in the last section we examine phrase order and elucidate some rules of song construction.

### Regional Variation

**Figure 2:**
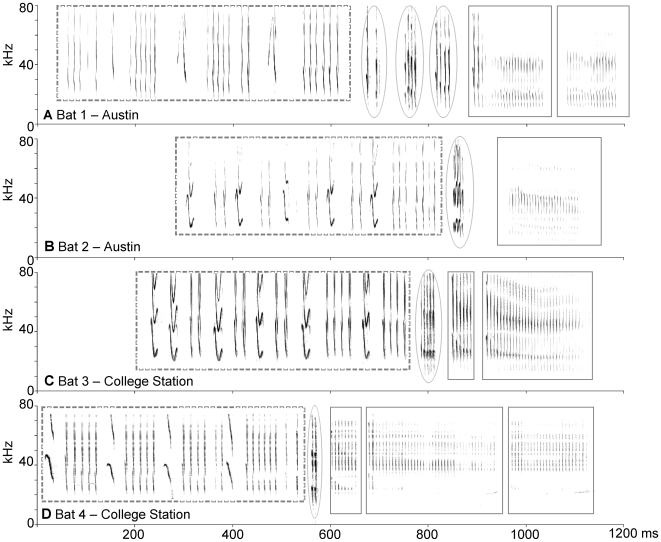
Spectrograms of songs from bats from the two regions. Two bats are from Austin (A and B) and two from College Station (C and D). Each chirp phrase is enclosed by a dashed rectangle, each trill is enclosed by an oval, and each buzz is enclosed by a solid rectangle. Time waveforms (Figure S1) and audio files (Audio S1–[Supplementary-material pone.0006746.s003] [Supplementary-material pone.0006746.s004] S4) of each of these songs are also provided. All songs are variants of the chirp-trill-buzz song type.

We first compared the phrase features at the two locations and found that the composition and structure of phrases did not differ (Table 1, *Phrase Variables*). Chirps were present in the songs of all males from both locations (Fig. 2). Chirp phrases at both locations had similar A and B syllables (Fig. 2), and there was no difference between the two locations in the number of A syllables that preceded each B syllable or in the number of B syllables per phrase (Table 1). The other two phrases, trills and buzzes, were also recorded from bats at the two locations. There was no difference in the number of syllables in either trills or buzzes between the two locations (Table 1).

**Table 1:** Mean±standard error and ANOVAs for phrase and song variables.

	Austin	CS	F^1^	*df*	*P*
**Phrase Variables**					
Chirp A syllables^2^	2.2±0.2	1.8±0.2	1.5	28	0.23
Chirp B syllables	5.6±0.5	5.4±0.6	0.1	24	0.82
Trill syllables	4.0±0.2	3.7±0.3	0.9	11	0.37
Buzz syllables	28.8±3.0	32.7±3.0	0.9	16	0.37
**Song Variables**					
Phrases	2.8±0.2	4.0±0.4	7.3	18	0.01
Phrases (>1 phrase)	3.7±0.2	4.1±0.3	0.9	16	0.42
Proportion with trills	0.5±0.1	0.7±0.1	3.7	17	0.07
Proportion with buzzes	0.4±0.1	0.8±0.1	9.7	17	0.006
Trills^3^	1.9±0.2	1.6±0.2	0.4	11	0.78
Buzzes^3^	1.8±0.1	2.0±0.2	0.4	12	0.49

1 all tests have 1 numerator degree of freedom.

2 number of chirp type A syllables per type B syllable.

3 songs without trills or buzzes excluded.

**Figure 3:**
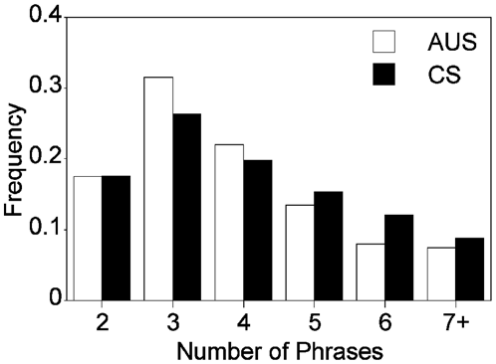
The distribution of song lengths at the two locations. The frequency of songs with 2 through 7 or more phrases at Austin (white bars, *N* = 200) and College Station (filled bars, *N* = 91). The distribution of song lengths is highly similar at the two locations.

Next we compared features of entire songs, (*eg* the total number of phrases, the number of buzzes etc.) at the two locations and found that song features were generally similar (Table 1, *Song Variables*). However, we found two minor regional differences. First, the proportion of songs with buzzes was greater in College Station (80%) than in Austin (40%; Table 1). Second, Austin bats emitted a greater number of songs composed only of chirps, i.e., single phrase songs, than did College Station bats (Table 1). In Austin, 37% of songs were chirp-only songs (*N* = 319 total songs) compared with only 2 % in College Station (*N* = 93 total songs). This resulted in a larger number of phrases per song in College Station compared to Austin (Table 1). When we excluded chirp-only songs from the analysis, there was no difference in the number of phrases per song between locations (Table 1), nor was there a difference between locations in the distribution of songs with different numbers of phrases (χ^2^ = 2.11, *P* = 0.84, *df* = 5, Fig. 3, chirp-only songs excluded). Thus, although we found a few minor differences in song composition, overall song construction and diversity are remarkably similar at the two locations.

**Figure 4:**
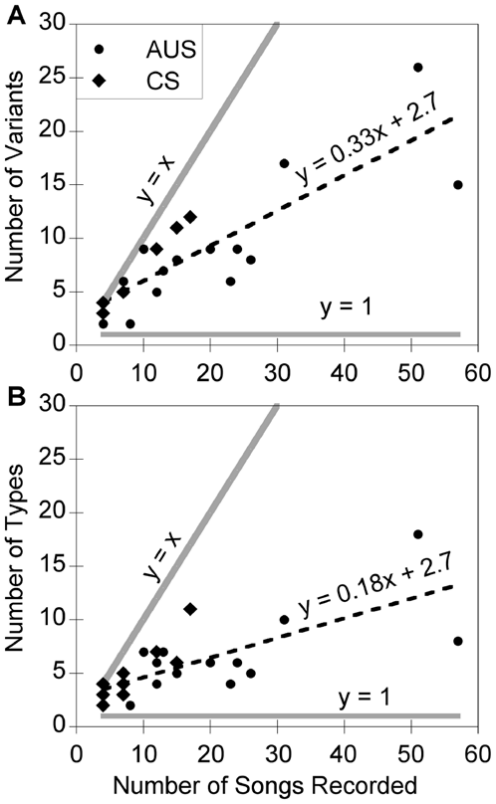
Song variants and song types. The number of song variants (with repetitions, A) and song types (without repetitions, B) as a function of the number of songs recorded for each bat. For each graph a line is shown for the case when every song recorded is unique (y = x) and when every song recorded is the same (y = 1). Only bats with a minimum of four recorded songs were included (*N* = 26 bats).

Next, we examined song diversity by comparing the number of song variants and song types per bat. Song variants and song types are defined by the sequence of phrases that compose a song either with (song variants) or without the inclusion of consecutive trill and buzz repeats (song types, see *Terminology* in Materials and Methods). We found that each location was not associated with only one or a few particular song variants or song types and that song diversity did not differ between locations. Out of the 412 songs we evaluated there were 87 different song variants and 36 different song types. The number of song variants per bat (ANCOVA, Fig. 4a, *F* = 27.0, *P*<0.0001, *df* all tests = 1, 22) and the number of song types per bat (Fig. 4b, *F* = 18.63, *P* = 0.0003) increased with the number of songs recorded. Neither the number of song variants (*F* = 1.17, *P* = 0.44) nor the number of song types (*F* = 1.32, *P* = 0.26) differed between locations. One caveat of these data is that we had fewer recordings for the majority of bats from College Station compared to Austin (Fig. 4).

### Within Individual Variation

Here we examined variation within individual bats and show that song features vary greatly from one rendition to the next. We evaluated variation relative to individuals, and renditions for eight of the same features that were used in the regional analysis (Table 1 and Table 2). We found much greater variation across renditions within individuals than among different individuals. In fact, 72–92% of variation occurred within individuals. However, the degree of variation across renditions was not consistent across all bats, as reflected by broad ranges in individual bat CV values (Table 2).

**Table 2:** Within individual (rendition) and between individual variation in phrase and song variables.

	N	Range	Region VCE	Bat VCE	Rendition VCE	Total CV	CV Range
**Phrase Variables**							
Chirp A syllables^1^	30	0–15	0.01	0.07	0.92	.99	0.38–1.67
Chirp B syllables	26	1–29	0.00	0.18	0.82	.69	0.11–0.81
Trill syllables	13	2–6	0.00	0.27	0.73	.26	0.09–0.40
Buzz syllables	18	8–65	0.00	0.28	0.72	.44	0.13–0.61
**Song Variables**							
Phrases	20	1–10	0.15	0.12	0.74	0.61	0.12–1.03
Phrases (>1 phrase)	18	2–10	0.00	0.12	0.88	0.41	0.12–0.57
Trills^2^	13	1–4	0.00	0.29	0.71	0.49	0.12–0.55
Buzzes^2^	14	1–4	0.00	0.16	0.84	0.43	0.28–0.65

For each feature the number of bats analyzed, the overall range in values (range), variance component estimates (VCE) that describe the proportion of variation attributable to region, bat, and renditions within bats, the overall coefficient of variation (Total CV), and the range in the coefficients of variation across bats (CV Range).

1 number of chirp A syllables per B syllable.

2 songs without trills or buzzes excluded.

Next, we investigated rendition variability by examining the number of song variants and the number of song types per bat. We found that bats produced multiple song variants and song types. This is illustrated by Figure 4, which shows that we continued to encounter novel song variants (Fig. 4a) and novel song types (Fig. 4b) as we sampled more songs. For comparison, we display two hypothetical lines. The first line at y = 1, represents the case where bats produce only one song variant and show no variation from one rendition to the next. The second line at y = x, represents the opposite extreme where every rendition of a song is unique. Our data lie between these two lines. Thus, bats did not sing a new variant or type in each rendition, nor did they produce the same song across all renditions. For song types the slope of the line was smaller than for song variants but still was between the two hypothetical lines (Fig. 4b). This shows that although variable repetitions of trills and buzzes contribute to song diversity, bats also vary broad scale patterns from one rendition to the next.

### Between Individual Variation

Finally we examined variation among different individuals. Although we found significant differences in song features across bats (nested ANOVA, *F*≥1.7, *P*<0.05, numerator *df* = 11–28, denominator *df* = 53–262, Table 2), these statistics were extremely sensitive because of large denominator degrees of freedom. Variation within bats was high and there was considerable overlap across bats. We also found considerable overlap in the use of song variants. Although each bat used a wide range of song variants, some of those variants were shared with many bats. For example, the three most common song variants, chirp, chirp-buzz, and chirp-buzz-buzz, were recorded from over 40% of the 33 bats. Sharing was even more pronounced with song types, that is with trill and buzz repetitions removed (Table 3). The most common song type, chirp-buzz, was produced by 70% of the males.

**Table 3:** The percentage of bats (*N* = 33) that produced the ten most common song types.

Song Type	% Bats	*N* Bats
chirp-buzz	70	23
chirp	55	18
chirp-trill-buzz	55	18
chirp-trill-chirp	42	14
chirp-trill	39	13
chirp-trill-chirp-buzz	21	7
chirp-buzz-chirp	15	5
trill-chirp	12	4
chirp-trill-chirp-trill	12	4
chirp-trill-chirp-trill-buzz	12	4

Note: the remaining 26 song types were produced by three bats or less.

### Phrase Order

In this section we examined the order of phrases in more detail. We found that songs were not constructed randomly. Instead, we identified several rules for song construction and particular phrase sequences that were greatly preferred over others. We examined phrase order at two levels: 1) phrase transitions or two-phrase combinations (*e.g.* chirp to trill, trill to chirp etc.) and 2) three-phrase combinations (*e.g.* chirp to trill to chirp, chirp to trill to buzz). For phrase transitions we tested if observed frequencies deviated from those predicted by a *random model*. For three-phrase combinations, in addition to the *random model* we tested if observed frequencies deviated from those predicted by a *first-order model* (see Methods). We then compared observed and expected frequencies and inferred general rules for song construction if observed phrase order frequencies 1) were close to 0 % or close to 100% and 2) deviated greatly from random predictions.

**Figure 5:**
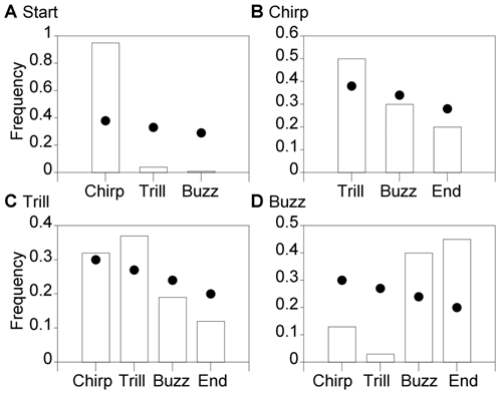
Observed and expected transition frequencies. Observed frequencies (bars) and expected frequencies (circles) of transitions from the start of song (A), chirps (B), trills (C) and buzzes (D) to each phrase or to the end of the song (“end”). For example, the first bar in A represents the observed frequency of beginning-chirp transitions. Expected frequencies were calculated in proportion to the relative abundance of phrases. Transitions were taken from all songs with greater than one phrase (*N* = 291 songs, 1,767 total transitions).

**Figure 6:**
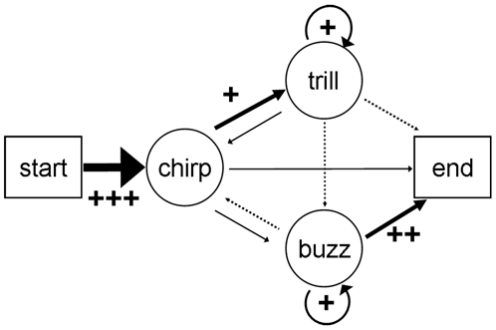
Model of song based on transition frequencies. Arrows represent transitions from one phrase to the next. Plus (+) symbols represent transitions that deviated from expected more than 10% (+), 20 % (++) or 50% (+++). Arrow thickness increases with transition frequencies based on values in Figure 5. No arrows were drawn for frequencies less than 0.05. See Figure 1 and Figure 2 for examples of each phrase.

We examined phrase transitions using all songs with more than one phrase (*N* = 291 songs). First, we found that transition frequencies deviated significantly from random expectations for the beginning of songs (χ^2^ = 407, *df* = 2, *P*<0.0001, *N* = 291) and for each song phrase (chirps: χ^2^ = 31.0, *df* = 2, *P*<0.0001, *N* = 445; trills: χ^2^ = 21.3, *df* = 3, *P* = 0.001, *N* = 392; buzzes: χ^2^ = 145, *df* = 3, *P*<0.0001, *N* = 348). Comparing observed and expected values revealed three major phrase-order rules: 1) songs begin almost exclusively with chirps (Fig. 5a and Fig. 6); 2) trills do not follow buzzes, but instead always follow chirps or another trill; and 3) the majority (90%) of buzzes are followed by another buzz or occur at the end of the song (Fig. 5d and Fig. 6). In fact, if a song contained a buzz it ended in a buzz 84 % of the time (155 of 185 songs with buzzes).

Next we examined if rules existed at higher levels, specifically for three-phrase combinations. For these analyses only songs with at least three phrases were used (*N* = 232 songs). We tested whether the frequency of three-phrase combinations deviated from two models: a simple *random model* and a 1^st^ order Markov model, the *first-order model* (see Methods). Indeed, three-phrase combination frequencies deviated from both models (*random model*: χ^2^ = 405, *df* = 21, *P*<0.0001; *first-order model*: χ^2^ = 182, *df* = 19, *P*<0.0001, *N* = 565, some three-phrase combination frequencies were pooled so that expected frequencies were greater than five). We observed all but one of 22 possible combinations and the most common and least common combinations matched model expectations. Furthermore, twenty of the 22 combinations deviated by only 4% or less from either model. Thus although three-phrase combinations are not randomly generated there are no simple rules for three-phrase combinations; instead song construction is quite variable.

**Figure 7:**
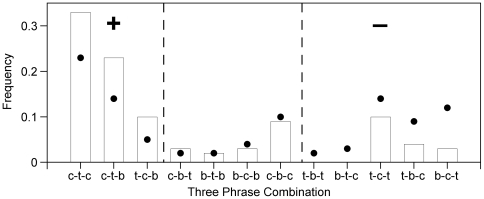
Observed and expected three-phrase combination frequencies. Observed frequencies (bars) and expected frequencies (circles) from the first-order model of three phrase combinations without trill or buzz repeats. “c” = chirp, “t” = trill and “b” = buzz. Combinations to the left of the dashed line occurred more frequently than expected (labeled with +) while combinations to the right of the dashed line on the right occurred less frequently than expected (labeled with –). Only songs with greater than three phrases were included (*N* = 183 songs, 302 total transitions).

Finally we examined three-phrase combinations on a broader scale by examining song types, that is after removing trill and buzz repeats. This simplified the analysis because it reduced the number of possible combinations from 22 to 12. We compared the frequency of three-phrase combinations to the *first-order model* since in the previous analysis it was the more complex model and was a closer fit to the data. We found that three-phrase combinations also deviated significantly from expectations (χ^2^ = 93.5, *df* = 11, *P*<0.0001, *N* = 302 transitions from 183 songs with three non-repeating phrases). When we examined observed frequencies we found that over 75% were comprised of only three of the twelve possible combinations: chirp-trill-chirp, chirp-trill-buzz and to a lesser extent trill-chirp-buzz (Fig. 7). In fact, all four songs in Figure 2 are examples of chirp-trill-buzz combinations.

## Discussion

In this paper we show that the songs of Brazilian free-tailed bats are hierarchically organized, have many highly stereotyped features, and follow specific syntactical rules, yet vary considerably within and between individuals. Many song features were highly conserved across all bats regardless of location and likely represent species-specific stereotypy. For example, all songs are composed of the same four types of syllables. In all songs, syllables are combined in a similar manner to form three common phrases. Furthermore, some aspects of song construction follow a few basic rules, while at higher-order levels a few broad scale patterns are clearly preferred. However, embedded in these design uniformities, we observed diversity in the detailed structures of songs among individuals and particularly across an individual's renditions.

In addition to quantifying overall song structure and variation, a major goal of this analysis was to draw parallels to the songs of other taxa. Our findings suggest that the songs of *T. brasiliensis* may be more analogous to those of some birds than to other mammals. In mammalian song, elements are combined non-randomly [13], [19]. However, specific structural rules like those we have observed in *T. brasiliensis* have rarely been reported in other mammal songs (but see [15], [18], [23]) even though they are commonly observed in birds [7], [8], [11]. Additionally, in bats [24] and mice [13], songs often proceed as trains of similar syllables that grade into other syllables, often with many intermediates. *T. brasiliensis*, on the other hand, produce highly stereotyped syllables that do not grade into each other, but instead are organized into discrete and distinct phrases much like the songs of many bird species.

One major advantage of the discrete way in which *T. brasiliensis* songs are constructed is that it greatly facilitates quantitative analyses. Discrete phrases permit categorization of songs into distinct “types” that parallels the birdsong literature. In this study, we used the term “song variant” for each unique combination of phrases, including different numbers of repeats of trills and buzzes. This classification scheme was quite narrow because songs were classified differently when they varied by a single trill or buzz repetition, even if they followed the same general pattern. However, removing repeats from the analysis created broader categories. For example in Figure 2 each of the four songs is a different song variant but all are the same song type. Thus, our song types are more analogous to avian “song types” that are used as an estimate of repertoire size and vocal complexity. We found that the number of types increased linearly with the number of songs recorded, instead of reaching an asymptote at maximal repertoire size. Clearly more recordings per individual are needed to obtain an estimate of the full extent of the song repertoire. However, we were able to compare song type usage and variation across individuals and regions. The differences between song variants and song types were also informative because they highlight one primary mechanism by which *T. brasiliensis* introduce variability into their song, by the selective repetition or subtraction of particular phrases.

We compared song features at two locations and found almost no geographical variation. Songs from Austin and College Station were remarkably similar. Indeed, some songs recorded at the two locations were virtually indistinguishable (e.g. Fig 2b and Fig 2c). Both populations used the same syllables and phrases to compose their songs, both included similar numbers of phrases and syllables per song, both followed the same global phrase order rules, and at both locations, individuals showed extensive and variable use of repeats within songs. Considering that at one location bats were recorded in their natural environment while the other was in captivity, these similarities are remarkable and suggest that the overall song structure is generally robust.

As mate attraction signals, we expected species-specific song features to facilitate the recognition of conspecifics. However within the context of a species-specific song template, regional variation is extremely common. Dramatic differences can occur over small geographic ranges in the form of culturally-transmitted dialects [25] while genetic differentiation can also contribute to song variation [3]. Our observation that there was a species-specific global template common to songs at both locations probably reflects the fact that *T. brasiliensis* is a genetically contiguous population that migrates across a broad geographical range [20]. Extensive dispersal and migration can prevent the development of geographic variation by either innate or genetic mechanisms. The two colonies studied here are separated by roughly one hundred miles. A previous analysis concluded that the population of free-tailed bats in and around College Station are a hybrid population composed of two separate subspecies of *T. brasiliensis* within Texas [26], including representatives of a migratory subpopulation found in central Texas, including Austin (*T.b. mexicana*) and a non-migratory population found in eastern Texas (*T.b. cynocephala*). Currently very little is known about the year-to-year roost fidelity of these bats or how large their foraging territories may spread over the course of a single season or throughout their lifetime. Thus, the lack of geographical variation we observed may be attributable to the potentially large areas over which these bats interact with each other.

Our results show that the primary source of variability in song construction came from between and within individual variation. Although as much as 30 % of the variation in song features was due to differences among bats, overlap was considerable, and even greater variation occurred across renditions within individuals. A similar pattern emerged when song types were examined. Some song types were shared across many bats but each bat also sang many different song types. The high degree of variability in songs sung by individuals coupled with the overlap in the usage of particular song types between individuals makes it unlikely that a single song feature can be used for distinguishing among individuals.

Our analysis of phrase order revealed three important characteristics of song construction in *T. brasiliensis*. First, *T. brasiliensis* songs follow three basic syntactical rules: 1) songs always begin with chirps, 2) trills do not follow buzzes and 3) buzzes predominately occur at the end of songs. Second, we discovered that at a broad scale, that is when consecutive repetitions are removed, only a subset of combinations occurs and particular combinations are preferred (chirp-trill-chirp, chirp-trill-buzz, and trill-chirp-buzz). Third, although on a broader scale a subset of combinations is disproportionately preferred, within any song, there could be a number of repeated trills or repeated buzzes that contribute to song diversity. Interestingly, similar patterns of syntax, particularly the use of specific orders and the addition or deletion of element repetitions have been documented in various bird species [27]–[30].

One aspect of *T. brasiliensis* song that has been observed in other bats is the use of buzzes. In sac-winged bats, Behr (2006) examined whether song features affected reproductive success. They concluded that the fundamental frequency and duration of long buzzes within songs were the best predictors of reproductive success because they provided females with honest information about the fitness of the singing male. Free-tailed bats use long buzzes in agonistic displays, in defense of territories and during physical confrontations with other males [22], and their appearance in songs may reflect a willingness to aggressively defend a territory. Communication buzzes were found to be significantly longer than the average feeding buzz emitted during echolocation in the field [22], indicating that there may be some selective force favoring longer buzzes when they are used for communication purposes by free-tailed bats. We would hypothesize that similar to the sac-winged bat the addition or subtraction of buzz phrases in free-tailed bat songs may reflect the current level of stamina or aggressive motivation of the individual. If so, it may be the case that the observed regional differences in the number of songs with buzz phrases could be the result of different social conditions of the colony members at the time of the recordings.

Our results overwhelmingly indicate that male free-tailed bats dynamically vary syllable number, phrase order, and phrase repetitions. One possible explanation for this diversity is female preference. In sac-winged bats, song complexity, measured by the number of unique syllable types, was positively correlated with the number of females a male had on his territory [5]. In many bird species females appear to prefer more complex and/or variable songs [31], [32]. Whether or not female free-tailed bats are attracted to more variable songs remains to be determined. Alternatively, song variation, particularly the diversity of song types we observed, may be produced in different behavioral contexts or have functionally different meanings. Support for functionally relevant syntax has been found in the chick-a-dee call system [33], [34]. Interestingly, these calls are similar to *T. brasiliensis* song in multiple ways, they both have relatively few building blocks (chickadee calls consist of four notes) that are used to create many combinations and they both follow simple rules of syntax that are elaborated upon with repetitions of particular notes [29], [30]. Future research should explore the role of female preference and social context on song variation and song type use in this species.

In conclusion, *T. brasiliensis* produce complex variable songs that are easily categorized and quantified. We present the first evidence that bats routinely vary songs across renditions via subtle shifts in syllable number, phrase repetition and/or phrase order. Bird song has been the basis for understanding the evolution of vocal complexity as well as the physiology of vocal production. This study provides a quantitative foundation for future research into a complex mammalian vocal signal particularly with respect to vocal plasticity and evolution.

## Materials and Methods

### Study site and animals

This study was conducted at two locations. The first was a captive colony of approximately 60 *T. brasiliensis* in Austin, Texas and a second wild colony at Texas A&M, in College Station, Texas. The Austin colony has been maintained by the author (BF) for ten years, and the identity, sex, and history for each individual has been documented. Bats were housed in a wooden structure measuring 4.9 m (length)×3.7 m (width)×3.7 m (height). Two windows allowed filtered sunlight to enter. Humidity was maintained at 60% or above and temperatures varied in the building from approximately 22 to 26 degrees C. Cloth-covered heating pads placed in cages during evening hours provided bats with the option of accessing temperatures reaching 29 degrees C. The bats roosted in fabric pouches positioned along the walls and ceilings of open wooden cages and had access to the entire building. Bats had continual access to water and beetle larvae (*Tenebrio molitor*), and were also offered a blended mixture of larvae, baby food, and vitamin supplements in the evening [4], [35]. At the Austin colony, we observed behaviors and recorded songs during the mating season (March and April) from 2003 to 2007. Males frequently emitted songs spontaneously during this time of year but we also induced singing by approaching territories with reproductive females.

At Texas A&M, groups of free-tailed bats were recorded from year-round natural colony of approximately 100,000 to 250,000 bats located within the university's athletic complex. Within the complex, small groups of bats that were reliably located in easily accessible places were videotaped and recorded once a week in the early afternoon (12:00–2:00 pm) for a 52-week period extending from January 2006 to January 2007, although songs were only detected between March and September. Some groups contained several singing bats, and some groups contained only one singing bat. Bats that produced songs were identified on videotaped recordings because they came to the front-most edge of the roost and performed a territorial display. Some these bats were captured and transferred to a bat vivarium in the Biology department for further behavioral studies. The vivarium consisted of two rooms (4×5×3 m^3^) that had regulated light-dark cycles adjusted with a light timer to mimic the natural external photoperiod. The two rooms were connected with a large sliding door that remained open to provide more room for flight. The rooms were temperature and humidity controlled. Within the vivarium bats roosted in artificially constructed bat houses (Maberry Centre Bat Houses, Daingerfield, TX). Bats were trained to feed themselves, and were fed a diet of mealworms supplemented with vitamins and essential fatty acids. All husbandry and experimental procedures were in accordance with NIH guidelines for experiments involving vertebrate animals and were approved by the local IACUC. In some instances individual male bats were recorded singing in the vivarium and in a soundproof recording chamber in the lab. Males and females were housed separately, but were not acoustically isolated.

### Acoustic Recordings

Vocalizations were recorded using a ¼-inch microphone (Brüel and Kjær type 4939) and custom-made amplifier. In 2003 and 2004 signals were recorded into a custom-made digital time expander. The time expander recorded a maximum of 1 second that was expanded to 10 seconds at 16 bits and was played onto a computer at a sample rate of 44.1 kHz. In 2005 and 2006, calls were recorded directly onto a computer at a sample rate of 300 kHz using a high-speed data acquisition card (National Instruments, NI PCI 6251 M Series, Austin, Texas, USA) and Avisoft Recorder Software (version 2.97, Avisoft Bioacoustics, Berlin, Germany). Both systems allowed recordings up to 150 kHz, well above the frequency content of vocalizations. At Texas A&M, ultrasonic vocalizations were recorded using an externally-polarized condenser microphone (Avisoft Bioacoustic, Berlin Germany, model CM16) and digitized at 250 kHz sampling rate using the Avisoft UltrasoundGate hardware (Avisoft Bioacoustics, model 116–200) for storage on a personal computer running the accompanying Avisoft-Recorder software v. 2.9. For analyses, we used all songs that were of sufficient quality for measurements and syllable identification.

### Terminology

We used the following terms to describe vocalizations:

*Song*: vocalizations emitted by males during the mating season that have multiple types of syllables and phrases. Songs were separated by intervals of silence of at least 115 ms (see *Defining Songs* below)

*Syllable*: the smallest acoustic unit of a vocalization. In this study it is equivalent to one continuous emission surrounded by silence of at least 1 ms. Equivalent to a note.

*Phrase*: a combination of one or more types of syllables that may be repeated in a song. *Simple phrases* are composed of one type of syllable. *Complex phrases* are composed of different types of syllables.

*Song Variant*: a unique sequence of phrases. For example the song variant in Figure 1 is: chirp-buzz-trill-trill-trill-buzz.

*Song Types*: a unique sequence of phrases that does not include consecutive trills and buzzes *i.e.* one or more buzzes are considered a single buzz and one or more trills are considered a single trill. For example the song type in Figure 1 is: chirp-buzz-trill-buzz

### Defining Songs

Frequently songs were produced in obviously discrete units. However, on other occasions songs were produced in bouts over longer periods of time and the beginnings and endings of multiple songs were difficult to determine. We used bout analysis [36] on the intervals between all syllables to objectively cut recordings into discrete songs. We measured the intervals between all syllables of all recordings (*N* = 19,614) using SIGNAL (v 4, Engineering Design) and calculated the log frequency of intervals per unit time across the range of interval lengths. We then used non-linear regression (PROC NLIN, SAS institute, Cary, North Carolina, USA) to fit a two-process model to the data where a “fast” process represented inter-syllable intervals and a “slow” process represented inter-song intervals [36]. This analysis resulted in a conservative interval threshold of 115 ms for the slow process with only 1% of intervals greater than threshold. This resulted in 412 songs, where each song was a continuous set of syllables with inter-syllable intervals less than 115 ms.

### Song Features

We used oscillograms and spectrograms to identify phrases within songs and syllables within phrases. All songs were composed of three easily identified phrases: chirps, trills and buzzes. We examined the composition of each phrase. For trills and buzzes we counted the number of syllables per phrase. We compared the number of syllables in trills and buzzes and the duration of trills and buzzes using paired t-test on one randomly selected buzz and one randomly selected trill from each bat that produced both phrase types (*N* = 23). For chirps we calculated the number of type B syllables per phrase and the ratio of type A syllables per type B syllable (see *Phrase Variables* Table 1). Next, we examined the composition of each song: the total number of phrases, the proportion of songs with trills, the proportion of songs with buzzes, the number of trills for songs with at least one trill, and the number of buzzes for songs with at least one buzz (See *Song Variables* in Table 1). Finally for each song recorded we assigned a song variant and song type based on its sequence of phrases.

### Regional and Individual Analyses

We examined variation in phrase and song variables relative to individuals and regions. Data were analyzed with nested mixed ANOVAs where individual bats were random factors nested within regions (PROC MIXED, SAS). We used restricted maximum likelihood (PROC VARCOMP, SAS) to calculate variance component estimates for region, individual, and rendition (residual within bat variation). The only exceptions were the proportion of songs with buzzes and the proportion of songs with trills, which were calculated per bat and only analyzed relative to region. To control for unevenness in sampling across bats for each variable we 1) only used bats with at least five songs and 2) for bats with greater than ten songs we randomly selected ten songs from each bat. To further examine variation relative to individuals, for each variable we calculated the coefficient of variation (CV = standard deviation/mean) for 1) the total sample and 2) each individual bat (presented as the range in CV values).

In addition to phrase and song features we examined the number of song variants and the number of song types per bat for bats in which we had at least four songs. The number of song variants and the number of song types increased linearly with sample effort (Fig. 4) indicating that larger sample sizes would be required to find the total repertoire size of individuals. We used ANCOVAs (analysis of covariance) on the number of song variants and song types with location as a factor and the number of recordings as a covariate. We first tested a full model with an interaction effect between recordings and location that determines whether the slopes of the two lines (one for each location) differ. However, the interaction effects were not significant (*P*>0.05) and so we removed this effect from the model.

### Phrase Order

The aim of this analysis was to determine if there are rules for constructing songs. To accomplish this we compared phrase order frequencies with those predicted from two models. Specifically, we used chi-squared tests to determine whether the frequency of 1) transitions from one phrase to the next (two-phrase combinations), and 2) three-phrase combinations, deviated from expected. For two-phrase combinations, expected values were calculated using the *random model*. For three-phrase combinations, in addition to the *random model*, we used a more complex model, the *first-order model*. With the *random model*, predicted frequencies of transitions and three-phrase combinations were based solely on the frequency of the constituent phrases. The *random model* is the simplest model; the likelihood of any phrase occurring is independent of the identity of any preceding phrases. The *first-order model*, on the other hand, was calculated from the abundance of transitions from one phrase to the next. It is a first-order Markov model and under this model, the likelihood of a phrase occurring depends on the identity of the previous phrase.

First we examined transitions using the *random model*. For the beginning of each song and for each phrase, we determined expected transitions to the following phrase or to the end of song. Thus, for calculations we treated the start and end of songs as “phrases” and so the first phrase of a song was considered the “second phrase” of a start transition. Expected transition frequencies were simply the expected frequencies of the second phrase: the number of times the second phrase (or end) was observed divided by the number of times all possible phrases were observed. By using “possible” phrases we incorporated the fact that some transitions were impossible, such as chirp-chirp and start-end.

Next we examined three-phrase combinations. For three-phrase combinations we did not include start and end positions. All three-phrase combinations were calculated for songs with at least three phrases. In essence we used a three-phrase sliding window. For example the song chirp-trill-chirp-buzz has two three phrase combinations: chirp-trill-chirp and trill-chirp-buzz. Using the *random model* expected three-phrase combination frequencies were calculated as the product of the frequency of each phrase. Again, this model assumes that the current phrase is independent of the previous phrase. For the *first-order model* expected three-phrase combinations were calculated as the frequency of the first phrase multiplied by the frequency of the first transition multiplied by the frequency of the second transition. Again, this model assumes that the current phrase depends on the probability of a transition from the previous phrase.

Although the primary goal of these analyses was to determine whether phrase order was non-random, they were also used to elucidate phrase-order rules, that is combinations or patterns that were highly non-random and either absent or ubiquitous. To this end we calculated deviations from expected as the difference between observed frequencies and expected frequencies. However, for three-phrase combinations, patterns were not obvious because most of the large number of possible combinations (22 for three-phrase combinations) were observed. Thus, in a final analysis we removed trill repeats and buzz repeats. This is the same as running analyses on song types instead of song variants. This reduced the number of possible three-phrase combinations to twelve, which we then compared to expected frequencies calculated from the *first-order model* described above.

All values are presented as means±standard errors unless stated otherwise.

## Supporting Information

Figure S1 Time waveforms normalized to a maximum of 1 volt, of the four bats presented in Figure 2.
(0.31 MB TIF)

Audio S1 Song of Austin Bat 1 slowed eight times.
(0.72 MB WAV)

Audio S2 Song of Austin Bat 2 slowed eight times
(0.72 MB WAV)

Audio S3 Song of College Station Bat 1 slowed eight times
(0.60 MB WAV)

Audio S4 Song of College Station Bat 2 slowed eight times
(0.34 MB WAV)

Movie S1 A male bat sings while performing a wing flapping display in front of his territory, a cloth pouch, where some females are roosting.
(2.48 MB MOV)

Movie S2 A slowed version of Movie S1 showing a male display and song.
(1.13 MB MOV)
