# Supplementary figures and images for: Versatility and Stereotypy of Free-Tailed Bat Songs
Source: PLoS One. 2009 Aug 25;4(8):e6746. doi: 10.1371/journal.pone.0006746 (PMC2727915; doi:10.1371/journal.pone.0006746)

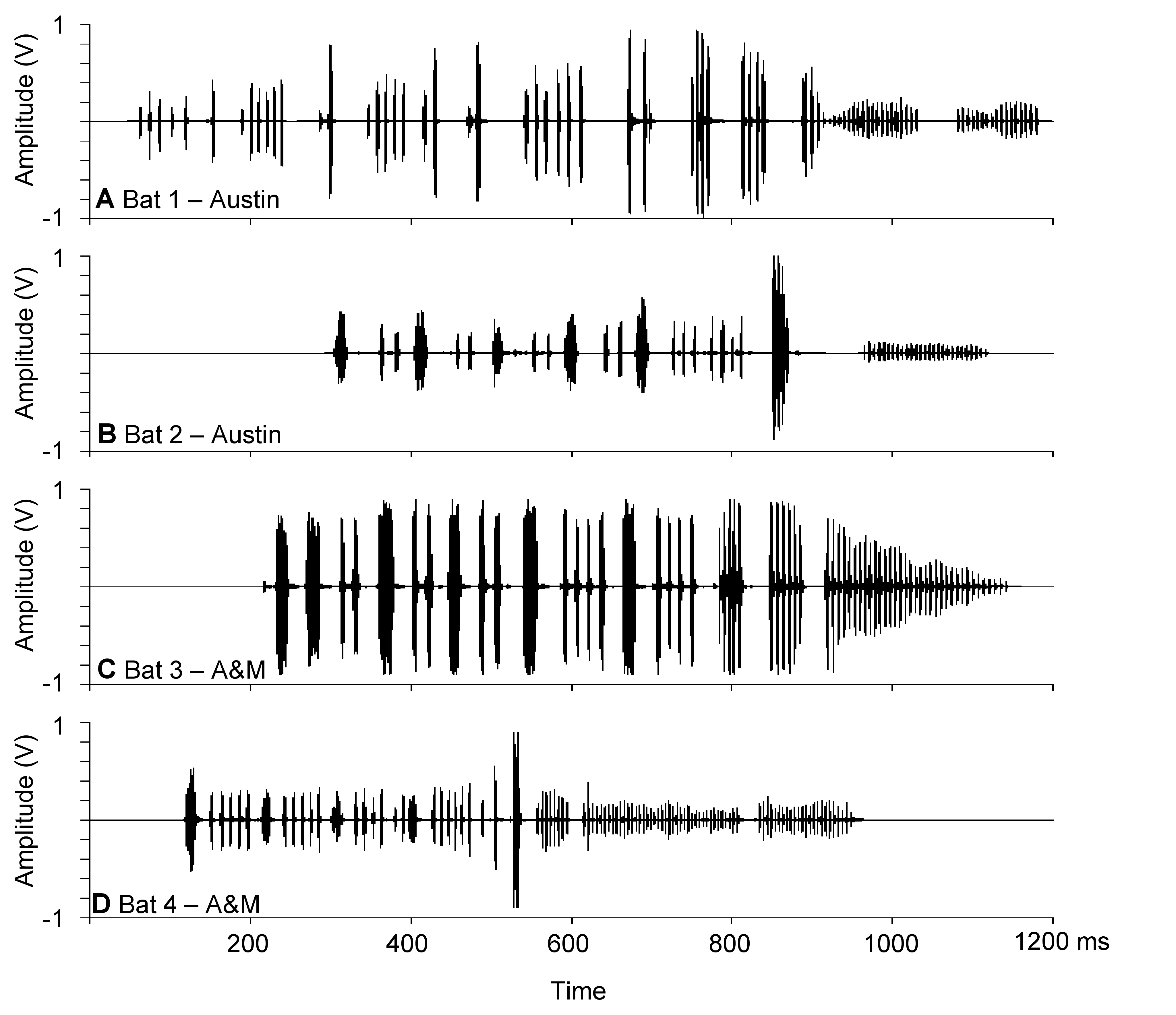

Supplement: Figure S1 — Time waveforms normalized to a maximum of 1 volt, of the four bats presented in Figure 2. (0.31 MB TIF) [file pone.0006746.s001.tif]
